# Geomagnetic Disturbances and Pulse Amplitude Anomalies Preceding M > 6 Earthquakes from 2021 to 2022 in Sichuan-Yunnan, China

**DOI:** 10.3390/s24134280

**Published:** 2024-07-01

**Authors:** Xia Li, Rui Qu, Yingfeng Ji, Lili Feng, Weiling Zhu, Ye Zhu, Xiaofeng Liao, Manqiu He, Zhisheng Feng, Wenjie Fan, Chang He, Weiming Wang, Haris Faheem

**Affiliations:** 1 State Key Laboratory of the Tibetan Plateau Earth System, Environment and Resources (TPESER), Institute of Tibetan Plateau Research, Chinese Academy of Sciences, Beijing 100101, China; lxqhdz@163.com (X.L.); qurui@itpcas.ac.cn (R.Q.); zhuweiling@itpcas.ac.cn (W.Z.); zhuye@itpcas.ac.cn (Y.Z.); haris@itpcas.ac.cn (H.F.); 2 Qinghai Earthquake Administration, Xining 810001, China; ynufll@sina.com; 3 University of the Chinese Academy of Sciences, Beijing 100049, China; 4 Sichuan Earthquake Administration, Chengdu 610041, China; dzj350102@163.com (X.L.); dzj763215@163.com (C.H.); tariwwm@163.com (W.W.); 5 Chongqing Earthquake Administration, Chongqing 401147, China; dzj435217@163.com; 6 Jiangsu Earthquake Agency, Nanjing 400071, China; fengzs2001@soho.com; 7 Yunnan Earthquake Agency, Kunming 650244, China; fan_095011106@126.com

**Keywords:** pulse amplitude method, geomagnetic disturbance, testbed station array, geomagnetic vertical intensity polarization, earthquake prediction

## Abstract

Compelling evidence has shown that geomagnetic disturbances in vertical intensity polarization before great earthquakes are promising precursors across diverse rupture conditions. However, the geomagnetic vertical intensity polarization method uses the spectrum of smooth signals, and the anomalous waveforms of seismic electromagnetic radiation, which are basically nonstationary, have not been adequately considered. By combining pulse amplitude analysis and an experimental study of the cumulative frequency of anomalies, we found that the pulse amplitudes before the 2022 Luding M6.8 earthquake show characteristics of multiple synchronous anomalies, with the highest (or higher) values occurring during the analyzed period. Similar synchronous anomalies were observed before the 2021 Yangbi M6.4 earthquake, the 2022 Lushan M6.1 earthquake and the 2022 Malcolm M6.0 earthquake, and these anomalies indicate migration from the periphery toward the epicenters over time. The synchronous changes are in line with the recognition of previous geomagnetic anomalies with characteristics of high values before an earthquake and gradual recovery after the earthquake. Our study suggests that the pulse amplitude is effective for extracting anomalies in geomagnetic vertical intensity polarization, especially in the presence of nonstationary signals when utilizing observations from multiple station arrays. Our findings highlight the importance of incorporating pulse amplitude analysis into earthquake prediction research on geomagnetic disturbances.

## 1. Introduction

The process of earthquake initiation and occurrence is accompanied by various degrees of electromagnetic signals, which can be caused by piezoelectric, piezomagnetic and kinetic effects [1,2,3]. Rock fractures when its stress exceeds its load-bearing capabilities and the rock is compressed. This causes the crystal lattice of the rock to become discontinuous, resulting in a jump in the electric potential and the release of electromagnetic wave signals [4]. Therefore, an effective extraction method can not only successfully extract significant anomalous signals but also deepen the understanding of the mechanism of seismomagnetic anomalies. In recent years, it has been shown that signal anomalies occur in ultralow-frequency (ULF) (0.001–10 Hz) electromagnetic waves during rock fracture [5,6]. Hao et al. (2003) [2] conducted experimental studies of the rock fracture process in zero magnetic space and found that ULF electromagnetic radiation was indeed generated during the rock fracture process and that short-period precursory changes in the magnetic field strength near the rock occurred near the time of fracture [2].

Regarding the extraction of short-period geomagnetic anomaly signals, the geomagnetic vertical intensity polarization method has a clear mechanism, is a mature processing method, is effective at extracting anomaly information (e.g., [7]), is the most widely used method internationally and is generally regarded as an accurate method of geomagnetic anomaly extraction [5,8], which can not only suppress interference from external source fields but also highlight magnetic anomalies in the source area. The current geomagnetic vertical intensity polarization method has also become a research hotspot and is used to study earthquakes, such as a series of moderate earthquakes in Kashgar from 2004 to 2007 [9], the 2009 Binchuan M5.0 earthquake [10], the 2017 Jiuzhaigou M7.0 earthquake and Jinghe M6.6 earthquake [11], the 2017 Arakan M5.0 earthquake [12], the 2017 Milin M6.9 earthquake [13], the 2021 Maduo M7.4 earthquake [14] and the 2022 Menyuan M6.9 earthquake [15,16], which were characterized by significant anomalous electromagnetic signals prior to the earthquake.

However, the above geomagnetic vertical intensity polarization method is calculated using the spectral method, which is applicable to smooth electromagnetic signals. In reality, the anomalous waveforms of seismic electromagnetic radiation are basically nonstationary signals such as pulses, sawtooth, step jumps, square waves, irregular sine waves, etc., which occupy a very short period during the analysis period, so the anomalous amplitude obtained by the traditional spectral method is seriously suppressed. In this study, we use the pulse amplitude method to calculate the geomagnetic vertical intensity polarization anomaly, which can greatly increase the anomaly amplitude. By tracking the information of the mobile observatory array of seismic magnetic disturbances in the eastern Sichuan-Yunnan junction and using the pulse amplitude method to calculate the geomagnetic vertical intensity polarization, a significant synchronous magnetic anomaly before the Luding M6.8 earthquake on September 5, 2022, is identified. We further investigate the spatial and temporal propagation characteristics of pre-earthquake magnetic anomalies in station arrays and provide new methods for the extraction of magnetic anomaly information for great earthquake prediction in this region.

## 2. Data and Methods

### 2.1. Geomagnetic Observation Data from the Sichuan-Yunnan Regional Testbed Station Array

Since the beginning of 2015, under the organization and arrangement of the earthquake monitoring and forecasting department of the China Earthquake Administration, the project team has been carrying out the standardization of seismic electromagnetic disturbance observation instruments to solve the critical problems of inconsistent observation standards, incomplete components, uneven performance and lack of frequency response curves of instruments found in previous investigations to achieve the ultimate goal of obtaining accurate and reliable observation data for earthquake prediction.

**Figure 1:**
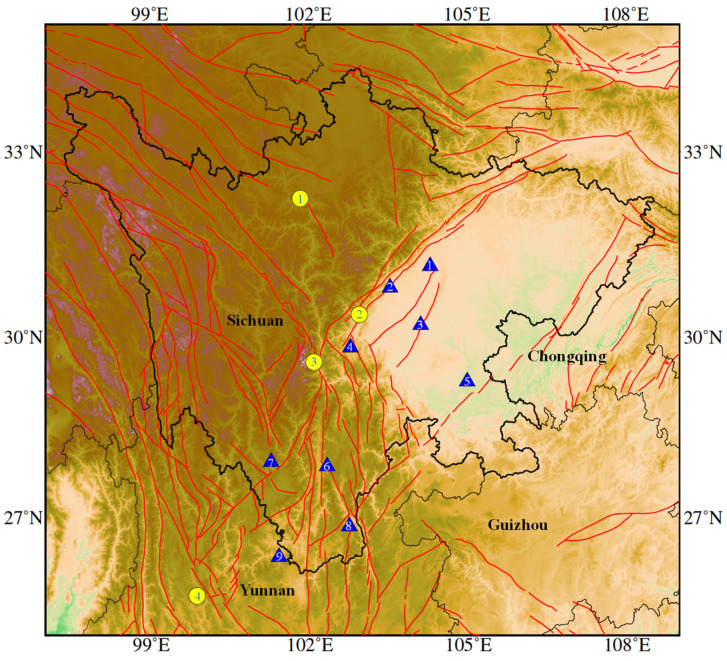
Tectonic map of the study area. The yellow circles indicate the M > 6 earthquakes in this region from 2021 to 2022 (1: 2022 Malcolm M6.0; 2: 2022 Lushan M6.1; 3: 2022 Luding M6.8; 4: 2021 Yangbi M6.4). The triangles represent the nine stations of the newly constructed Sichuan-Yunnan regional testbed station array used in this study (1: Deyang; 2: Chongzhou; 3: Renshou; 4: Xingjing; 5: Fushun; 6: Xichang; 7: Muli; 8: Shimenkan; 9: Nanshan).

To further verify the long-term stability of the performance of China’s seismic electromagnetic disturbance observation instruments, as well as the ability to observe short-term seismic anomalies, the China Earthquake Administration has organized the construction of the Sichuan-Yunnan regional testbed station array, which consists of nine stations and a data center. The nine stations are the Xichang 03 Well, Xingjing Station, Chongzhou Station, Muli Geomagnetic Observatory, Fushun Station, Nanshan Station, Deyang 08 Well, Renshou Station and Shimenkan Geomagnetic Point (Figure 1).

Since the normal operation of the Chuan-Yunnan Test Array in 2021, the instruments at each site have produced daily three-component vertical geomagnetic observations in the X (north), Y (east) and Z (vertical down) directions, at a sampling rate of 100 Hz. During the trial period, the M6.0 earthquake on 16 September 2021, in Luxian County occurred approximately 35 km from the array, and the M6.0 earthquake on 6 April 2022, in XingWen, Sichuan Province, occurred approximately 100 km from the array. During the trial period, an M6.0 earthquake in Lu County, Sichuan Province, on 16 September 2021; an M5.1 earthquake in XingWen, Sichuan Province, on 6 April 2022; and an M6.4 earthquake in YangBi, Yunnan Province, on 21 May 2021, also occurred at a distance of approximately 35 km from the array, and short-term anomalies were recorded in the array’s data prior to the earthquakes, as analyzed by forecasting techniques.

### 2.2. Methods

The geomagnetic vertical intensity polarization method includes the following steps:

(1) $$R_{zh}=\left|\frac{Z(\omega )}{H(\omega )}\right|$$

(2) $$H(\omega )=\sqrt{H_{x}^{2}(\omega )+H_{y}^{2}(\omega )}$$

where *Z*(*ω*) is the spectral amplitude of the geomagnetic vertical component, *H*(*ω*) is the spectral amplitude of the full vector of the geomagnetic horizontal component, *H_x_*(*ω*) is the north–south-oriented spectral value of the geomagnetic horizontal component, *H_y_*(*ω*) is the east-west-oriented spectral value of the geomagnetic horizontal component, and *ω* is the circular frequency.

However, the anomalous waveforms of seismic electromagnetic radiation basically manifest as nonstationary signals such as pulses, sawtooth, step jumps, square waves and irregular sinusoids on smooth recording curves and are characterized by the fact that the duration of the anomalous waveform is very short during the analysis period. The anomaly amplitude obtained by the traditional spectral method is severely suppressed. Zeng et al. (2011) [17] analyzed the geomagnetic changes at the Chengdu seismic station before the 2008 Wenchuan M8.0 earthquake by calculating the amplitude of changes per minute in the second sampling data of the magnetic fluxgate, which showed an anomalous change of up to 20 nT in the vertical component and 10 nT in the horizontal component in the 2 days prior to the M8 earthquake. The algorithm has the advantage of highlighting the magnitude of seismic anomalies compared to spectra, which is referred to as the pulse amplitude method for convenience.

Therefore, for the spectral method, the components of *Z*(*ω*), *H_x_*(*ω*) and *H_y_*(*ω*) in the geomagnetic vertical intensity polarization are changed to the amplitudes *A_z_*, *A_Hx_* and *A_Hy_* within a certain window length, and

(3) $$A_{ZH}=\left|\frac{A_{Z}}{A_{H}}\right|$$

(4) $$A_{H}=\sqrt{A_{Hx}^{2}+A_{Hy}^{2}}$$

where *A_z_*, *A_Hx_* and *A_Hy_* are the variation amplitudes of the vertical, northward and eastward components, respectively, in the calculation window.

Based on the observation bandwidth of the station array instrumentation, the data sampling interval is 0.01 s, the polarization ratio, i.e., the polarization value, is calculated, and the result is a sequence of seconds.

## 3. Results

### 3.1. Pre-Earthquake Geomagnetic Anomalies of the 2022 Luding M6.8 Earthquake

**Figure 2:**
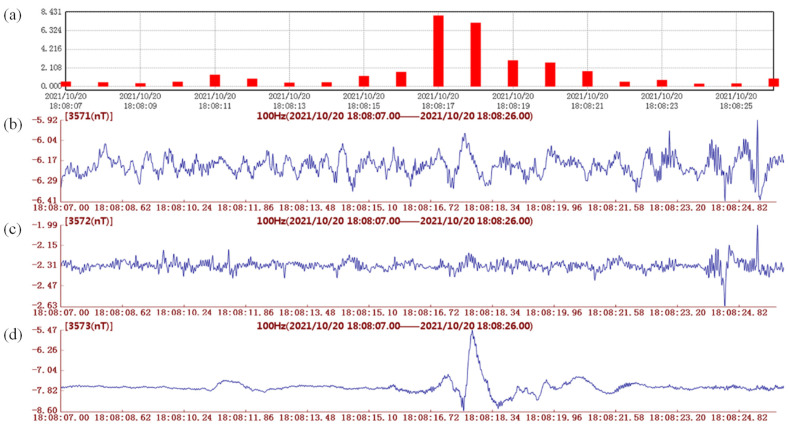
Geomagnetic anomalies observed on November 20, 2021. (**a**) Geomagnetic vertical intensity polarization value (*y*-axis, this study) with a precision of seconds; (**b**–**d**) geomagnetic vertical intensity polarization value (*y*-axis, this study) with a precision of 0.01 s: (**b**) northward; (**c**) eastward; (**d**) vertical.

The magnetic anomaly signals related to the underground seismic source area were extracted by tracking the data from the testbed station array in the eastern part of the Sichuan-Yunnan junction and using the geomagnetic vertical intensity polarization method (Figure 2 and Figure S1). According to the characteristics of the seismic activity level in Sichuan Province and neighboring areas, the seismic magnetic perturbation pulse amplitudes were analyzed during periods of earthquake quiescence. The results show that the pulse amplitude anomalies at each station are lower during earthquake quiescence periods, and the cumulative frequency is also relatively low, which is used as a background field variation reference. To recognize the anomalous information more intuitively and uniformly, based on the results of the analysis of several groups of earthquake cases, we set the threshold value of the polarization of the geomagnetic vertical intensity to 10 to eliminate low polarization ratios, and the cumulative number of days with polarization values exceeding the threshold value was fixed, as shown in Table 1, which was used as the criterion for anomalous discrimination.

**Figure 3:**
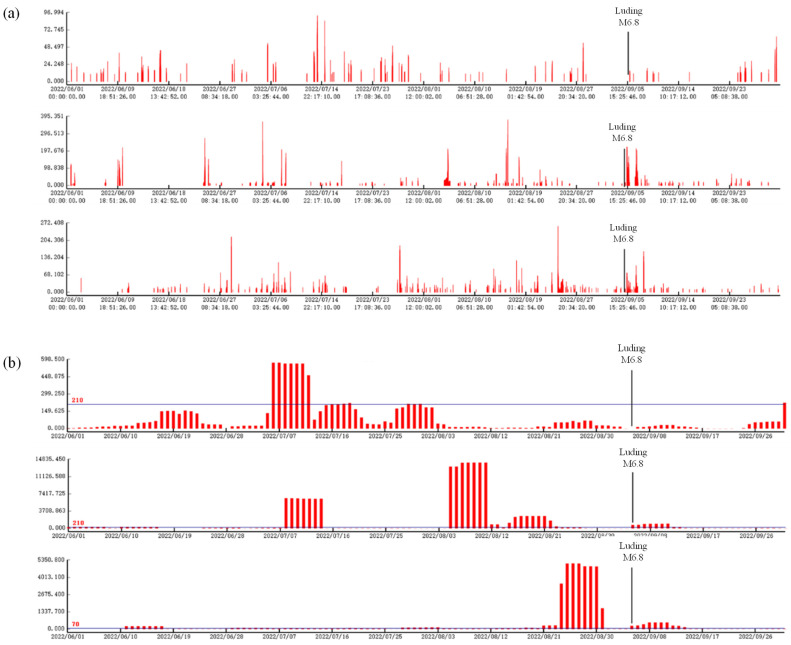
Geomagnetic vertical intensity polarization values (*y*-axis, this study) of the Sichuan-Yunnan station array before the Luding M6.8 earthquake on 5 September 2022. (**a**) Geomagnetic vertical intensity polarization pulse amplitude variations. Upper: Xichang station; middle: Xingjing station; lower: Deyang station. (**b**) Cumulative number of times per day that the pulse amplitude exceeds the threshold value. Upper: Xichang station; middle: Xingjing station; lower: Deyang station.

By combining these data, we found that there were significant magnetic anomalies in the surrounding observation stations before the 2022 Luding M6.8 earthquake, which are described as follows:

(1) The Xichang station had had a high frequency of geomagnetic vertical intensity polarization since July 6, and a polarization maximum of 90 was reached from 13 July to 15 July, which was approximately four times greater than the background field value of the earthquake quiescence period; this high-frequency change continued for 61 days. The Luding M6.8 earthquake occurred, and the high-frequency change in polarization after the earthquake exhibited attenuation characteristics (Figure 2a), with an epicentral distance of 204 km. The statistical results of the cumulative number of days with a polarization value exceeding the threshold (Figure 2b) exceeded the set threshold value of 210, and the high values lasted for approximately 13 days, showing characteristics of obvious fluctuations and a decrease in changes before the earthquake from 6 July 2022, onward.

(2) Half a year before the earthquake, the irregularly high polarization of the vertical intensity of the geomagnetic field at the Xingjing Station appeared to be concentrated in clusters, and the polarization was not high during the 59 days after the 2022 Luding M6.8 earthquake. Because of the close proximity to the epicenter of the earthquake (only 69 km), the effect of the earthquake was significant, and the large change in the polarization value lasted for three days. Then, the high polarization value gradually decreased to normal on 8 September (Figure 3a); the cumulative number of days with polarization values exceeding the set threshold continued to increase for approximately 13 days. The cumulative number of days with polarization values exceeding the threshold (Figure 3b) lasted for nearly 24 days. The subsequent Luding M6.8 earthquake tended to reverse these changes in magnitude for 8 days after the earthquake.

(3) At the Deyang station, the polarization value of the geomagnetic vertical intensity fluctuated significantly approximately three months before the earthquake, and the cumulative number of days with a polarization value exceeding the threshold exceeded the set threshold value. The high values lasted for 11 days, and the subsequent Luding M6.8 earthquake, with an epicentral distance of 286 km, resulted in a continued change in the high polarization values for 11 days; the change in the high values tended to return to normal thereafter. The specific parameter information is shown in Table 2.

Before the Luding M6.8 earthquake, the pulse amplitude and cumulative frequency results of the three stations mentioned above, excluding the low values at the respective stations, showed the characteristics of multiple-station synchronization of anomalies, and the pulse amplitudes were either the highest observed or among the highest values in the analyzed period. The group synchronization changes were consistent with the previous understanding of magnetic anomalies, generally showing high synchronization values before the earthquake and gradual recovery after the earthquake. Therefore, this kind of variation in signal characteristics is likely a pre-earthquake magnetic anomaly and a short-term earthquake precursor index.

### 3.2. Pre-Earthquake Geomagnetic Anomalies of the 2021 Yangbi M6.4 Earthquake

**Figure 4:**
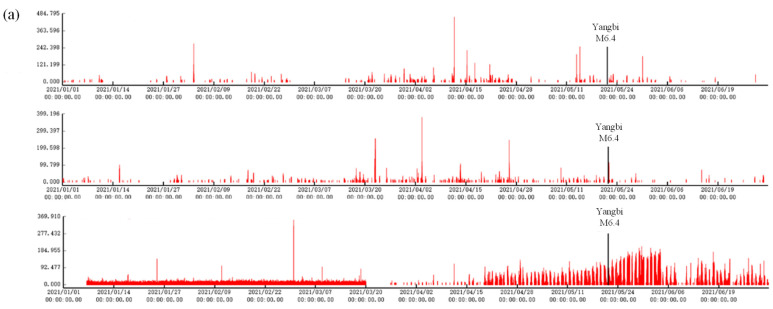

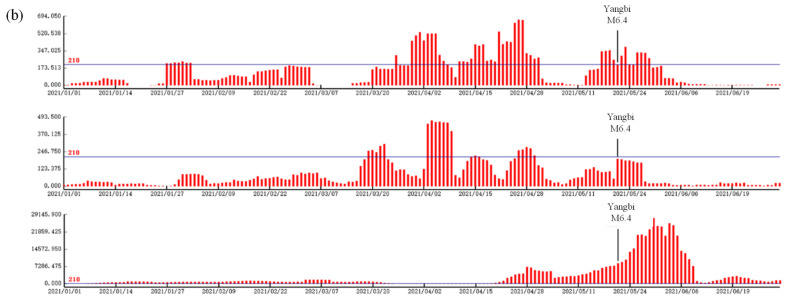
Geomagnetic vertical intensity polarization values (*y*-axis, this study) of the Sichuan-Yunnan station array before the 2021 Yangbi M6.4 earthquake on 21 May 2021. (**a**) Geomagnetic vertical intensity polarization pulse amplitude variations. Upper: Xichang station; middle: Muli station; lower: Nanshan station. (**b**) Cumulative number of times per day that the pulse amplitude exceeds the threshold value. Upper: Xichang station; middle: Muli station; lower: Nanshan station.

Several stations experienced similar variations in geomagnetic disturbance data before the 2021 Yangbi M6.4 earthquake (Figure 4 and Table 3):

(1) The Xichang station exhibited a pattern of increasing polarization values after the beginning of 2021, and the cumulative daily number of polarization values exceeding the threshold exhibited significant increases and decreases from 29 March onward, with a sustained high value for approximately 35 days and then a high value again after 12 days of quiescence. The high values lasted for a total of 53 days, and then the high values decreased to normal 7 days after the earthquake, with the epicenter at a distance of 346 km.

(2) At the Muli station, the polarization value increased beginning on 19 March 2021, and the cumulative daily number of polarization values exceeded the threshold, after which the anomaly pattern decreased until it returned to a low value after the earthquake, and the epicentral distance reached 284 km.

(3) The Nanshan station showed an increasing trend in polarization since 20 April 2021, and the pattern of change continued for approximately half a month after the earthquake. The cumulative daily number of polarization values exceeding the threshold lasted for 31 days. The values decreased to normal 15 days after the earthquake, and the distance from the epicenter was 202 km.

### 3.3. Pre-Earthquake Geomagnetic Anomalies of the 2022 Lushan M6.1 and 2022 Malcolm M6.0 Earthquakes

**Figure 5:**
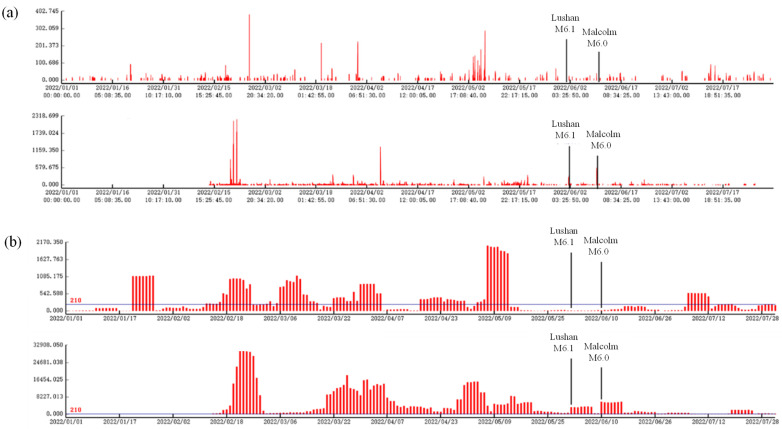
Geomagnetic vertical intensity polarization values (*y*-axis, this study) before the 2022 Lushan M6.1 and 2022 Malcolm M6.0 earthquakes. (**a**) Geomagnetic vertical intensity polarization pulse amplitude variations. Upper: Xichang station; lower: Fushun station. (**b**) Cumulative number of times per day that the pulse amplitude exceeds the threshold value. Upper: Xichang station; lower: Fushun station.

Anomalies also existed before the 2022 Lushan M6.1 and 2022 Malcolm M6.0 earthquakes according to the calculation of data from the eastern Sichuan-Yunnan junction (Figure 5 and Table 4):

(1) The frequency and amplitude of polarization at the Xichang station have increased since 21 January 2022, and the cumulative daily number of polarization values exceeding the threshold also appears to have exceeded the threshold for a long time; additionally, the distances between the epicenters are 318 km and 478 km.

(2) Fushun station has experienced large changes in polarization values since 17 February 2022, and the cumulative daily number of polarization values exceeding the threshold also appears to be high during this period. The fluctuation of high values continued until approximately one week after the earthquake when it tended to recover.

Therefore, the changes in the polarization values reflecting the high magnetic disturbance before the M > 6 earthquakes exhibited temporal and spatial synchronization, and the patterns exhibited some similarities.

## 4. Discussion

The significance of reliable earthquake predictions for society has been recognized for many decades, but uncertainties regarding source initiation, rupture phenomena, and the accuracy of both the timing and magnitude of earthquake occurrences have often been difficult to overcome [18]. The conventional method in seismology is based on the analysis of large and small historical earthquakes and attempts to identify patterns in the spatial and temporal distributions of earthquakes along given faults [19], which can only provide statistical estimates burdened by wide uncertainty margins [20,21]. However, as nonseismic signals and strong geomagnetic anomalies have been observed to occur repeatedly before large earthquakes (e.g., [22,23,24]), and due to the great sensitivity of geomagnetic field data anomalies to seismic activity (e.g., [25,26]), the seismic electromagnetic method, which plays an essential role in identifying preseismic anomalies as earthquake precursors (e.g., [27,28,29,30]), is regarded as one of the primary methods for earthquake prediction. As a potential precursor of earthquakes, seismo–electromagnetic phenomena could be used to predict the variability of the detected intensity, frequency, spatial, and temporal distribution around the epicenter. First, electromagnetic emissions in a wide frequency spectrum ranging from kHz to MHz are produced by opening cracks, which could be considered precursors of general fractures [31]. In terms of the predicted time of earthquakes, the magnetic field changes related to the Ms7.1 Loma Prieta earthquake that occurred on 18 October 1989, as reported by Fraser-Smith et al. (1990) [22], were among the most well-known ULF seismomagnetic phenomena, and they found that the amplitude of the ULF magnetic fields, especially at a frequency of 0.01 Hz, began to increase significantly approximately two weeks before the main shock [32]. Uyeda et al. (2002) [33] reported very robust results for seismo–electromagnetic phenomena and reported that the ratio of the electric potential between two different observation dipoles at a frequency of 0.01 Hz clearly exhibited anomalous changes approximately two months before the earthquake swarm. Some researchers have discovered increases in ultralow-frequency magnetic pulse activity starting two weeks before nearby seismic events and disappearing after the event [34]. Moreover, geomagnetic anomalies provide very important support for the prediction of earthquake location and magnitude. Earthquake-related magnetic signals may decrease with epicentral distance and increase with the magnitude of an earthquake event (e.g., [35,36,37,38]), suggesting that augmenting earthquake forecasting with magnetic anomalies might involve distance and magnitude dependencies [32].

However, due to solar activity, atmospheric disturbance, artificial noise, and other factors, signals representing geomagnetic anomalies are often difficult to distinguish from background noise [39]. Understanding how to highlight the signals representing geomagnetic anomalies from the source area and suppress interference from the external field is the key to extracting the signals of geomagnetic disturbances and anomalies before an earthquake. Cummer (2000) [40] proposed that the ionosphere plays a role in radio propagation that varies strongly with frequency, and at low frequencies, the ground and the ionosphere are good electrical conductors and form a spherical Earth–ionosphere waveguide. The Coronal Mass Ejection (CME) and Corotating Interaction Region (CIR, including geomagnetic storms) are the two main causes of international and geomagnetic disturbances in the solar–Earth space, and CME and CIR events both have durations as large as a scale of >10 min (e.g., [41]). One possible reason for the short period of preseismic geomagnetic disturbance is that the geomagnetic waves excited by different magnetic layers, including the aurora zone, have concordant durations of <100 s (e.g., [42]). Among the various excited geomagnetic waves, the Pc1 wave has the shortest duration (0.2–5 s), which might account for the observed ultralow-frequency pulsation of the geomagnetic field in this study [42]. We used the noise reduction process of the China Geomagnetic Observatory Network to eliminate the ULF Pc1 geomagnetic pulsations, i.e., the ground signatures of electromagnetic ion-cyclotron magnetospheric waves, through several indices for data evaluation of the reference background noise. This process is designed according to the distribution characteristics of international magnetic quiescence and disturbance days, as well as in different time periods, and its rationality will be verified by observation data (e.g., [11,43,44]). By comparing the reference background noise during the midnight period (with the most active excited geomagnetic wave noise) and the reference background noise during the quiescence period by using the first-order difference method, the indices effectively reduced most of the noise from excited geomagnetic waves (e.g., Pc1 waves), monitored the quality of observation data, timely reflected the problems existing in geomagnetic observatories, and were used to assess the minimum resolution of the geomagnetic observation network (e.g., [45]). In this study, we attempt to focus on the high-frequency band (0.5–10 Hz) of ULF waves (0.001–10 Hz), i.e., geomagnetic pulsations with durations of 0.1~2 s (Figure 2), instead of the traditional 5~100 s bands (the accuracy range of the traditional spectrum method). To calculate the intensity of vertical polarization, the reduction in excited geomagnetic wave noise is necessary for geomagnetic pulsation analysis, even in the mid-to-low latitude region (e.g., [46,47]).

By studying the emission characteristics of a ULF source within the Earth (0.01 to 100 Hz) and the propagation characteristics of such radiation to the surface of the Earth, the ionosphere, and the magnetosphere, numerical simulation results have shown that the vertical amplitude of the primary magnetic signal from the Earth’s crust is greater than or close to the horizontal amplitude [48]; that is, the ratio of the vertical amplitude to the horizontal amplitude of the magnetic field from the Earth’s crust is greater than or close to 1 (e.g., [49]). The measurements of ULF magnetic noise during a large earthquake (Ms = 7.1) in Guam on 8 August 1993 (depth ∼60 km), with a polarization analysis (Z/H) of the polarization ratio (ratio of horizontal to vertical magnetic field components) [8], have become an effective technique for extracting possible seismo–ULF emissions, even when signals from geomagnetic activity exist (e.g., [10,12,14,50,51,52,53,54,55,56,57,58]). Hobara et al. (2004) [59] analyzed ULF magnetic field emissions and observed three large earthquakes that occurred at different locations and suggested that seismic events may be associated with anomalous changes in the polarization ratio.

Based on second-sampled observation data from the FHDZ-M15 geomagnetic observation system, He et al. (2017) [60] extracted ULF geomagnetic anomalies prior to several medium or strong earthquakes. The vertical intensity polarization anomalies before the Jiuzhaigou Ms7.0 earthquake on 8 August 2017 were also identified [58]. The geomagnetic stations near the epicenter synchronously exhibited polarization above the threshold anomalies. Using multistationary anomalies, Feng et al. (2022) [49] further reported that Mw ≥ 6 earthquakes generally occur after anomalies occur, within a period ranging from 3 months to 1 year, from 2015 to 2021 in Qinghai, China. However, these studies, employing the spectrum method in vertical intensity polarization anomalies, have strong limitations in that the frequency bands cover only 5–100 s, while the bands of 0.1–5 s and >100 s are overlooked. In this regard, our simplified pulse amplitude method for calculating the intensity of vertical polarization can fill the gap in the research on the high-frequency ends (0.5–10 Hz) of ULF wave bands (0.001–10 Hz).

Additionally, many studies have shown that the polarization ratio significantly increased approximately several months before an earthquake (e.g., [52,61,62,63]). However, the method of geomagnetic vertical intensity polarization analysis (Z/H) is based on spectral analysis, which has smoothing and suppressing effects on the extraction of irregular electromagnetic radiation anomalies and is unfavorable for the extraction of anomalous information. This method is impacted by the quality of observation data and the deployment spacing of stations, and the signals representing geomagnetic anomalies typically manifest at only a single station close to the epicenter [39,64]. Hence, the reliability of abnormal signals is difficult to judge, and it is challenging to exclude data interference factors. Instead, the pulse amplitude method in this study can extract high-value anomalies with durations of 0.1~2 s. This method highlights the anomaly amplitude and is conducive to identifying and extracting signals representative of high-band ULF magnetic disturbances, providing more possibilities for earthquake prediction through geomagnetic anomalies.

## 5. Conclusions

Based on geomagnetic disturbance testbed station array observations at the eastern Sichuan-Yunnan junction since 2021, we used the geomagnetic vertical intensity polarization pulse amplitude method to extract magnetic disturbance and anomaly information before M > 6 earthquakes in Sichuan-Yunnan, and the following conclusions were drawn:

(1) The pulse amplitude and cumulative frequency results before the 2022 Luding M6.8 earthquake show multiple synchronous anomaly characteristics, and the pulse amplitude is the highest (or higher) during this period. Similar synchronization anomalies are characterized before the 2021 Yangbi M6.4 earthquake, 2022 Lushan M6.1 earthquake, and 2022 Malcolm M6.0 earthquake; these anomalies also show a pattern of migrating toward the epicenters with time.
(2) The synchronized changes are consistent with the previous understanding of magnetic anomalies, and all of these changes show the general characteristics of high pre-earthquake values and gradual recovery after the earthquake.
(3) The geomagnetic vertical intensity polarization pulse amplitude method for extracting geomagnetic vertical intensity polarization anomalies is effective, and this method can be applied to short-range earthquake prediction.

## Figures and Tables

**Table 1:** Geomagnetic vertical intensity polarization (GVIP) pulse amplitude anomaly thresholds.

Number	Station	GVIP Pulse Amplitude Threshold	Threshold of Cumulative Frequency of Anomaly (/day)	Duration Threshold (day)
1	Deyang	10	70	10
2	Chongzhou	10	70	10
3	Renshou	10	210	10
4	Xingjing	10	210	10
5	Fushun	10	210	10
6	Xichang	10	210	10
7	Muli	10	210	10
8	Shimenkan	10	210	10
9	Nanshan	10	210	10

**Table 2:** Anomaly occurrence parameters of the stations before the 2022 Luding M6.8 earthquake.

Station Name	Epicentral Distance (km)	Anomaly Start Time	Time Interval (day)	Threshold	Cumulative Frequency (/day)	Duration (day)
Xichang	204	6 July 2022	61	10	210	10
Xingjing	69	8 July 2022	59	10	210	10
Deyang	286	21 August 2022	15	10	70	10

**Table 3:** Anomaly occurrence parameters of the stations before the 2021 Yangbi M6.4 earthquake.

Station Name	Epicentral Distance (km)	Anomaly Start Time	Time Interval (day)	Threshold	Cumulative Frequency (/day)	Duration (day)
Xichang	346	29 March 2021	53	10	210	10
Muli	284	19 March 2021	63	10	210	10
Nanshan	202	20 April 2021	31	10	210	10

**Table 4:** Parameters of the stations before the 2022 Lushan M6.1 and 2022 Malcolm M6.0 earthquakes.

Earthquake	Station Name	Epicentral Distance (km)	Anomaly Start Time	Time Interval (day)	Threshold	Cumulative Frequency	Duration (day)
Lushan earthquake	Xichang	318/478	21 January 2022	131	10	30	10
Malcolm earthquake	Fushun	232/420	17 February 2022	104	10	30	10

## Data Availability

Data are available in the figures and tables of the manuscript or from the authors upon request.

## Supplementary Materials

The following supporting information can be downloaded at: https://www.mdpi.com/article/10.3390/s24134280/s1, Figure S1: Geomagnetic anomalies observed on 10 March 2023. (a) Geomagnetic vertical intensity polarization values (y-axis, this study) with precision in 1 s; (b–d) geomagnetic vertical intensity polarization values (y-axis, this study) with precision in 0.01 s: (b) northward; (c) eastward; (d) vertical.
