# Supplementary material for: Geomagnetic Disturbances and Pulse Amplitude Anomalies Preceding M > 6 Earthquakes from 2021 to 2022 in Sichuan-Yunnan, China
Source: Sensors (Basel). 2024 Jul 1;24(13):4280. doi: 10.3390/s24134280 (PMC11244273; doi:10.3390/s24134280)
Supplement: Supplementary file 1 [file sensors-24-04280-s001.zip › sensors-2962018-supplementary.pdf]

Supplementary Materials for

Geomagnetic Disturbances and Pulse Amplitude Anomalies Preceding M > 6 Earthquakes from 2021 to 2022 in Sichuan-Yunnan, China

Xia Li et al.

This PDF file includes:

Figure S1

Figure S1

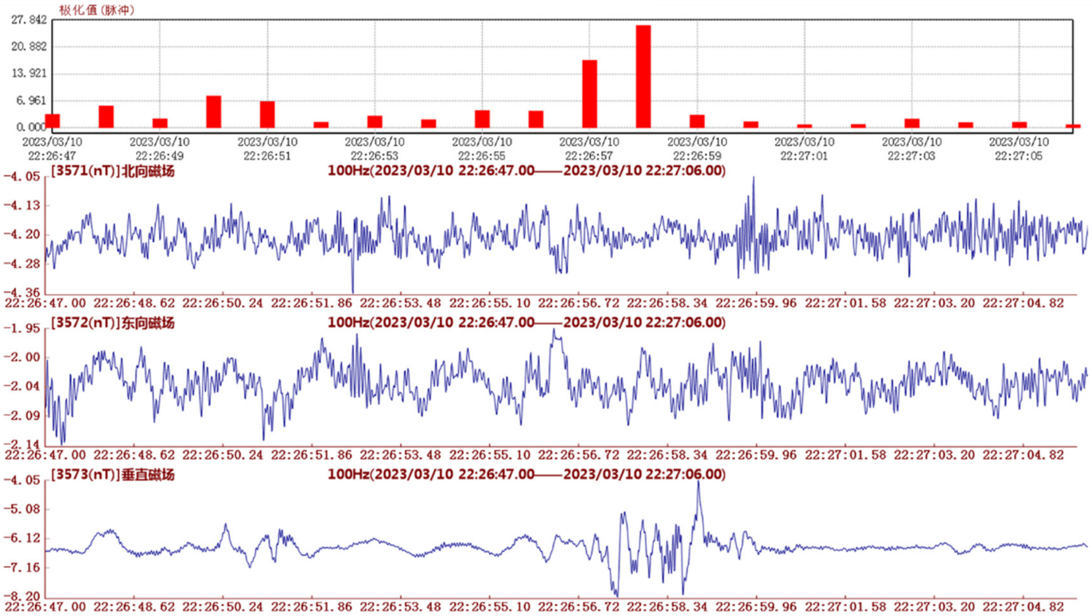

Figure S1. Geomagnetic anomalies observed on March 10, 2023. (a) Geomagnetic vertical intensity polarization values (y-axis, this study) with precision in 1 second; (b-d) geomagnetic vertical intensity polarization values (y-axis, this study) with precision in 0.01 s: (b) northward; (c) eastward; (d) vertical.
